# Role of microsurgical tumor burden reduction in patients with breast cancer brain metastases considering molecular subtypes: a two-center volumetric survival analysis

**DOI:** 10.1007/s11060-024-04728-w

**Published:** 2024-06-03

**Authors:** Jacopo Bellomo, Anna Maria Zeitlberger, Luis Padevit, Vittorio Stumpo, Meltem Gönel, Jorn Fierstra, Nathalie Nierobisch, Regina Reimann, Isabell Witzel, Michael Weller, Emilie Le Rhun, Oliver Bozinov, Luca Regli, Marian Christoph Neidert, Carlo Serra, Stefanos Voglis

**Affiliations:** 1 Department of Neurosurgery, Clinical Neuroscience Center, University Hospital and University of Zurich, Frauenklinikstrasse 10, 8091 Zurich, Switzerland; 2 Department of Neurosurgery, Cantonal Hospital St, Gallen, St. Gallen, Switzerland; 3 Department of Neuroradiology, Clinical Neuroscience Center, University Hospital and University of Zurich, Zurich, Switzerland; 4 Institute of Neuropathology, Clinical Neuroscience Center, University Hospital and University of Zurich, Zurich, Switzerland; 5 Department of Gynecology, University Hospital and University of Zurich, Zurich, Switzerland; 6 Department of Neurology, Clinical Neuroscience Center, University Hospital and University of Zurich, Zurich, Switzerland

**Keywords:** Breast cancer, Brain metastasis, Residual tumor, Extent of resection, Targeted therapy, Overall survival

## Abstract

**Background:**

Advancements in metastatic breast cancer (BC) treatment have enhanced overall survival (OS), leading to increased rates of brain metastases (BM). This study analyzes the association between microsurgical tumor reduction and OS in patients with BCBM, considering tumor molecular subtypes and perioperative treatment approaches.

**Methods:**

Retrospective analysis of surgically treated patients with BCBM from two tertiary brain tumor Swiss centers. The association of extent of resection (EOR), gross-total resection (GTR) achievement, and postoperative residual tumor volume (RV) with OS and intracranial progression-free survival (IC-PFS) was evaluated using Cox proportional hazard model.

**Results:**

101 patients were included in the final analysis, most patients (38%) exhibited HER2-/HR + BC molecular subtype, followed by HER2 + /HR + (25%), HER2-/HR- (21%), and HER2 + /HR- subtypes (13%). The majority received postoperative systemic treatment (75%) and radiotherapy (84%). Median OS and intracranial PFS were 22 and 8 months, respectively. The mean pre-surgery intracranial tumor volume was 26 cm^3^, reduced to 3 cm^3^ post-surgery. EOR, GTR achievement and RV were not significantly associated with OS or IC-PFS, but higher EOR and lower RV correlated with extended OS in patients without extracranial metastases. HER2-positive tumor status was associated with longer OS, extracranial metastases at BM diagnosis and symptomatic lesions with shorter OS and IC-PFS.

**Conclusions:**

Our study found that BC molecular subtypes, extracranial disease status, and BM-related symptoms were associated with OS in surgically treated patients with BCBM. Additionally, while extensive resection to minimize residual tumor volume did not significantly affect OS across the entire cohort, it appeared beneficial for patients without extracranial metastases.

**Supplementary Information:**

The online version contains supplementary material available at 10.1007/s11060-024-04728-w.

## Introduction

Breast cancer (BC) is the most frequent cancer affecting females, and the second most common cause of brain metastases (BM). In total, around 30% of patients with metastatic BC will develop BM in the course of their disease [1, 2].

Therapeutic strategies for breast cancer brain metastases (BCBM) encompass surgical resection, radiotherapy, and systemic pharmacotherapy. The efficacy of surgery for single BM and controlled systemic disease was established decades ago [3, 4], but its role in the era of molecularly guided systemic therapies is less explored. As a result, current indications for BM resection are limited by morphologic criteria, typically restricted to lesions greater than 3 cm in diameter and symptomatic ones, without considering tumor biology [5, 6]. In contrast to radiosurgery, microsurgical resection offers the advantage of promptly alleviating neurological symptoms caused by large lesions and obtaining tissue samples for histopathological diagnosis in cases of unknown primary malignancy [7]. Particularly, when immune-modulating therapy is part of the oncologic treatment plan, surgical resection can quickly relief edema, allowing for postoperative steroid tapering and reducing steroid-related toxicity [5, 8].

Several factors have been associated with an improved prognosis in BCBM patients, including patient’s age [9, 10], performance status at BM diagnosis, time interval between primary tumor and BM diagnosis [9, 10], number of BM lesions [9, 10], control of extracranial disease [11], and primary tumor molecular subtype [12]. In this context, the role of the extent of resection (EOR) or postoperative residual tumor volume (RV) on overall survival (OS) have been debated. Since BM represents one potential manifestation of systemic disease, cerebral local treatment may not fully determine the patient’s oncologic course [5, 13, 14].

In the present study, we investigated the association of EOR, achievement of gross-total resection (GTR) and RV with OS and intracranial PFS in a bicentric cohort of patients with BCBM who underwent microsurgical resection.

## Methods

### Study cohort, data acquisition and ethical considerations

All patients who underwent their first microsurgical resection of intraparenchymal BCBM between January 2012 and December 2022 at the Department of Neurosurgery, University Hospital Zurich (USZ) and between January 2013 and December 2021 at the Department of Neurosurgery, Cantonal Hospital St. Gallen (KSSG) were included in this analysis. Baseline characteristics and clinical outcomes during follow-up were retrospectively extracted for each patient from the respective institution’s prospectively recorded registries, approved by the local ethics review boards (Zurich identifier: PB-2017–00093; KSSG identifier: 2023–01343). Follow-up time was defined as the period from microsurgical resection to last follow-up or death. Baseline characteristics included BC molecular subtype, symptoms of BM at diagnosis, preoperative Karnofsky performance status (KPS) [15], total number of BM lesions, presence of extracranial metastases at BM diagnosis (as a proxy for systemic disease control), preoperative systemic treatment or radiotherapy, and time intervals between primary tumor and BM diagnosis, between diagnosis of metastatic disease and BM diagnosis, and between BM diagnosis and surgery. The BC molecular subtype was assessed through routine immunohistochemistry (IHC) assay conducted by the Institute of Neuropathology of each treatment center. Human epidermal growth factor receptor-2 (HER2) positivity was defined as a 3 + score in the IHC assay [16]. Hormone receptor (HR) positivity was defined as > 10% tumor cell nuclei showing reactivity to estrogen receptor staining in the IHC assay [17]. Clinical outcomes during follow-up included death and intracranial disease progression, defined as radiologically confirmed local or distant progression of cerebral metastatic disease, including the new detection of leptomeningeal spread. Additionally, we documented whether patients underwent systemic treatment, postoperative radiotherapy, and any additional local interventions, such as radiotherapy (besides initial postoperative radiotherapy) or re-craniotomy, during follow-up. If available, the cause of death was categorized as intracranial disease progression, systemic disease progression, or non-tumor associated, as previously described [18].

## Volumetric analysis

Radiological BM characteristics were assessed using pre- and postoperative contrast-enhanced and native T1-weighted magnetic resonance imaging (MRI) sequences. In cases where MRI was unavailable, computed tomography (CT) images were used. A single experienced neurosurgeon at each treatment center (USZ: SV; KSSG: AMZ) evaluated the images to determine total number of BM lesions and to assess pre- and postoperative total intracranial tumor volume, encompassing all intracranial lesions. Tumor volumes (in cubic centimeters, cm^3^) were semi-automatically segmented on T1-weighted gadolinium-enhanced images or contrast-enhanced CT images, utilizing iPlan Net (Brainlab AG, Munich, Germany). The EOR was calculated as [1—(postoperative total intracranial tumor volume of all residual lesions/preoperative total intracranial tumor volume) × 100]. GTR was defined as the 100% removal of the total intracranial tumor volume [19].

## Statistical analysis

All data processing and analysis steps were performed using R Studio (version 2023.09.1, R Studio Inc.) with open-source libraries. Initially, we investigated the association between EOR, GTR and RV with both OS and intracranial PFS. Kaplan–Meier survival curves were generated, and differences were evaluated using the log-rank test. Uni- and multivariable Cox proportional hazard models were utilized to determine hazard ratios (HR) and corresponding 95% confidence intervals (CI). Covariates included age at BM diagnosis, BC molecular subtype, neurological symptoms related to BM, total number of BM lesions, and the presence of extracranial metastases at BM diagnosis. Postoperative systemic and local treatments were not included to prevent time-dependent bias and possible uncontrollable confounding sources. Subsequently, subgroup analyses were conducted, stratifying by BC HER2 status and presence of extracranial metastases at BM diagnosis. Missing values were treated as missing at random and thus excluded from the respective analyses. Two-sided *p* values < 0.05 were considered statistically significant.

## Results

### Study cohort characteristics

We retrospectively identified 106 patients with BCBM who underwent neurosurgical resection, 74 at USZ and 32 at KSSG. Five patients were excluded because of previous operations on BM. Baseline characteristics are summarized in Table 1. The mean (SD) age at BM diagnosis was 56 (13) years. All but one patient were female. The majority (38%) exhibited HER2-/HR + BC molecular subtype, followed by HER2 + /HR + (25%), triple negative (TN; 21%), and HER2 + /HR- subtypes (13%). Median [IQR] time intervals between BC and BM diagnosis, metastatic disease diagnosis and BM diagnosis, as well as BM diagnosis and surgery were 44 [21, 96] months, 0 [0, 30] months, and 6 [3, 18] days, respectively. Symptomatic BM was present in 89% of patients, and 38% had extracranial metastases. Eighty-nine patients had fewer than five BM lesions, of whom 53 showed only one lesion. Median [IQR] follow-up was 16 [8, 36] months.

**Table 1:** Study cohort characteristics

	N = 101
Age at BM diagnosis, mean (SD)	56 (13)
Sex (%)	
- Female - Male	100 (99) 1 (1)
Preoperative KPS, median [IQR]	80 [70, 90]
BC molecular subtype (%)	
- HER2 + /HR + - HER2 + /HR- - HER2-/ HR + - HER2-/HR- (TN) - NA	25 (25) 13 (13) 38 (38) 22 (21) 3 (3)
BM symptoms (%)	
- Symptomatic - Asymptomatic	90 (89) 11 (11)
Time (in months) between BC and BM diagnosis, median [IQR]	44 [21, 96]
Time (in months) between metastatic disease diagnosis and BM diagnosis, median [IQR]	0 [0, 30]
Time (in days) between BM diagnosis and DOS, median [IQR]	6 [3, 18]
Extracranial metastatic lesion(s) at BM diagnosis (%)	
- Yes - No - NA	38 (38) 60 (59) 3 (3)
Number of metastatic lesion(s) (%)	
- 1 - 2–5 - > 5	53 (52) 36 (36) 12 (12)
Preoperative intracranial tumor volume [cm^3^], mean (SD)	26 (26)
Preoperative systemic treatment (%)	
- Yes - No	60 (59) 41 (61)
Preoperative radiotherapy (%)	
- Stereotactic radiotherapy - Whole-brain radiotherapy	4 (4) 3 (3)
EOR, mean (SD)	88 (22)
GTR (%)	
- Achieved - Not achieved - NA	38 (38) 58 (57) 5 (5)
Postoperative intracranial tumor volume [cm^3^], mean (SD)	3 (6)
Follow-up time (in months), median [IQR]	16 [8, 36]

BC, breast cancer; BM, brain metastasis; DOS, date of surgery; EOR, extent of resection; GTR, gross total resection (defined as EOR = 100%); HER2, human epidermal growth factor receptor-2; HR, hormone receptor; IQR, interquartile range; KPS, Karnofsky performance status; NA, not available; SD, standard deviation; TN, triple negative tumor

### Perioperative systemic and local treatments

**Fig. 1:**
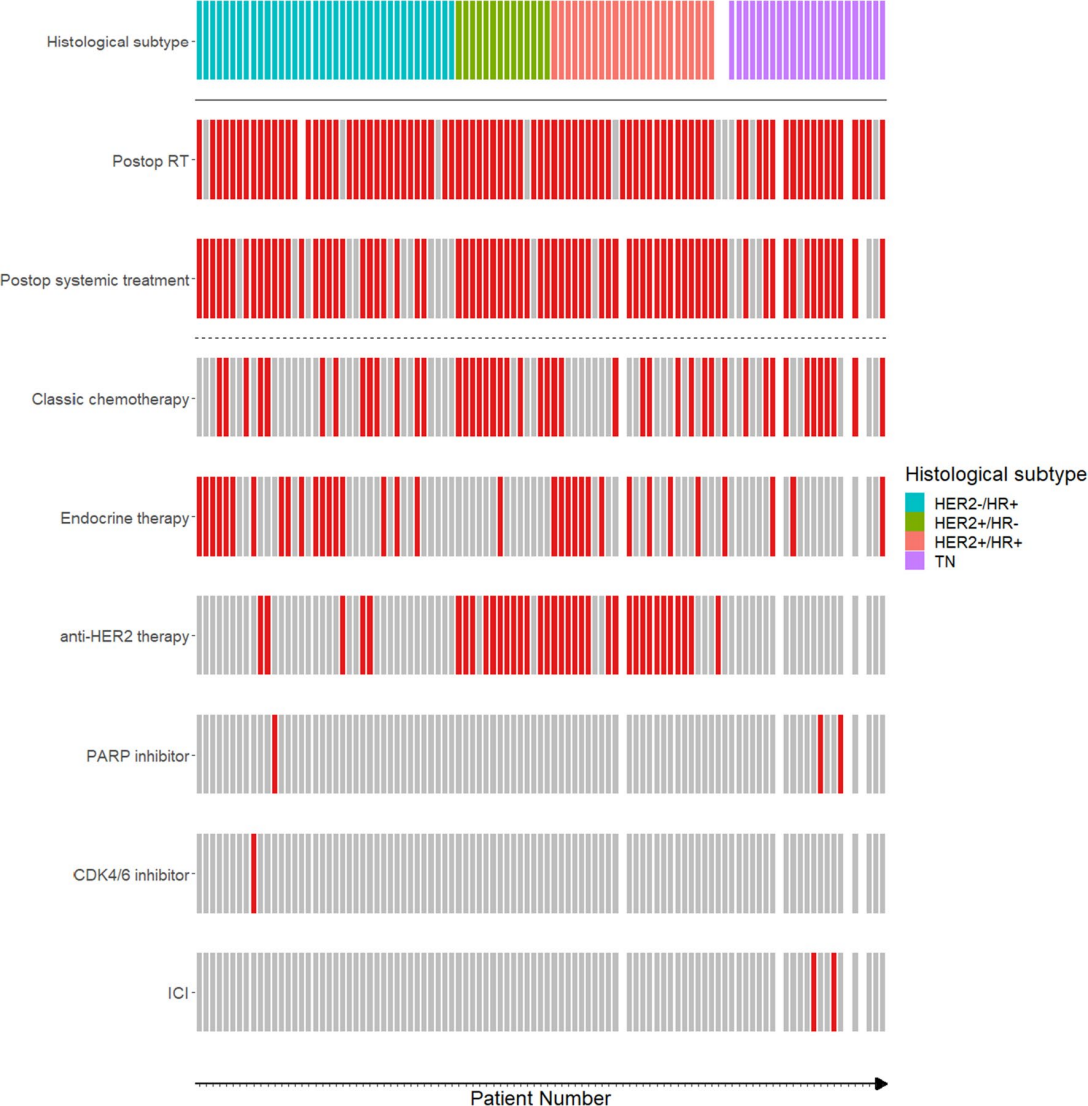
Molecular subtypes and postoperative therapies per patient. This plot indicates the molecular subtype per patient and the corresponding local or systemic therapies which the patient underwent after resection of the BCBM. HER2, human epidermal growth factor receptor-2; HR, hormone receptor; ICI, immune checkpoint inhibitor; RT, radiotherapy

**Fig. 2:**
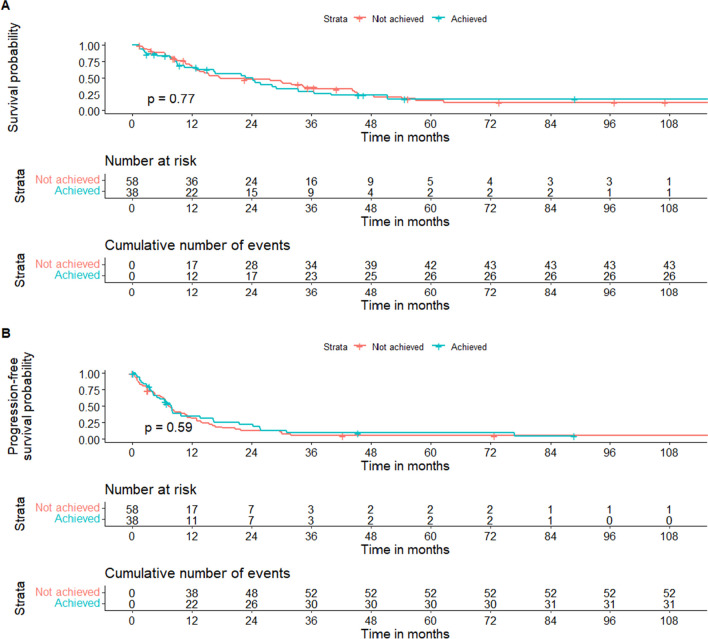
Overall survival and intracranial progression-free survival according to the achievement of GTR. Kaplan–Meier curve with log-rank statistic of patients stratified by the achievement of GTR, defined as EOR = 100%. **A** Overall survival, **B** intracranial progression-free survival. Time is reported in months from surgery; Follow-up time is clipped at 9 years for better readability. EOR, extent of resection; GTR, gross-total resection

At the time of surgery, 59% patients were on systemic BC therapies, while 7% had undergone preoperative radiotherapy for the BM lesion (Table 1). Postoperatively, 75% received systemic treatment, varying by BC molecular subtype: cytotoxic chemotherapy (45%; e.g. alkylating agents, anthracyclines, taxanes, platinum-based antineoplastics, vinca alkaloids, nucleotide analogs, inhibitors of topoisomerase I/II), anti-HER2 therapy (36%), endocrine therapy (34%), CDK4/6, immune checkpoint (ICI) and PARP inhibitor therapy (each < 5%, Supplemental Table 1 and Fig. 1). Postoperative radiotherapy was administered to 84% of patients, with 45% undergoing stereotactic radiosurgery (SRS), 27% receiving whole-brain radiotherapy (WBRT), and 12% having both (Supplemental Table 1 and Fig. 1). During follow-up, 44% of patients underwent at least one additional local intervention, including WBRT, SRS, re-craniotomy, or a combination thereof (Supplemental Table 1 and Fig. 2).

### Pre- and postoperative total intracranial tumor load

Ninety-nine patients underwent preoperative MRI, while two had CT. Postoperatively, 88 patients underwent MRI, and 13 had CT. The mean (SD) preoperative total intracranial tumor volume was 26 (26) cm^3^. The mean (SD) RV was 3 (6) cm^3^, with a mean (SD) EOR of 88% (22). GTR was achieved in 38 patients (38%, Supplemental Fig. 1). An EOR higher than 90% was achieved in 65 patients (64%). Among patients with a single BM lesion, the mean (SD) RV was 1 (4) cm^3^, and 32 of 52 (62%) achieved GTR. Further details on RV and EOR by BM lesion count and molecular subtype are shown in Supplemental Fig. 2 and 3. Patients with HER2 + /HR + and HER2-/HR + tumors showed higher RV and lower EOR (Supplemental Fig. 3).

### Survival analyses: OS and intracranial PFS

The median OS was 22 months (95% CI, 16 to 30) for all patients (Supplemental Fig. 4), 41 months (95% CI, 23 to 64) for patients with BC HER2-positive status and 14 months (95% CI, 11 to 23) for HER2-negative tumors (Supplemental Fig. 6). The median intracranial PFS was 8 months (95% CI, 6 to 10) for all patients (Supplemental Fig. 5). At last follow-up, 67 patients experienced radiologically confirmed intracranial disease progression: 8 had a local recurrence around the resection cavity, 26 had distant recurrence (including leptomeningeal spread), and the remaining 33 had a combination of local and distant recurrence. Of the 74 patients who died, the cause of death was available for 50 patients: 32 patients died of intracranial disease progression, 15 of systemic disease progression, and 3 of non-tumor associated causes.

The results of the Cox proportional hazard model analysis for OS are summarized in Table 2. EOR, GTR achievement, RV, and number of BM lesions were not associated with OS in either the univariable or multivariable models. However, patients with BC HER2-positive status had significantly longer survival, while the presence of extracranial metastases at BM diagnosis and symptomatic lesions were associated with shorter OS. Younger age showed a positive trend for longer OS, but it was not statistically significant. In subgroup analyses (Supplemental Tables 2–3), higher EOR (HR 0.62, 95% CI 0.41 to 0.94, p = 0.024) and lower RV (HR 1.57, 95% CI 1.03 to 2.39, p = 0.035), but not GTR achievement (HR 1.32, 95% CI 0.58 to 3.01, p = 0.50), were associated with longer OS in patients without extracranial metastases at BM diagnosis. No association of EOR, GTR achievement, and RV with OS was found in the subgroups of patients with BC HER2-positive and HER2-negative status, or in those with extracranial metastases.

**Table 2:** Cox regression analysis of the association between postoperative tumor volume load and overall survival

	Univariable		Multivariable					
	HR (95% CI)	*P* value	HR (95% CI)	*P* value	HR (95% CI)	*P* value	HR (95% CI)	*P* value
Extent of resection (EOR)#	0.98 (0.76 to 1.26)	0.60	0.89 (0.66 to 1.23)	0.50				
Gross-total resection (GTR)								
- Not achieved - Achieved	- 1.07 (0.66 to 1.74)	0.80			1.53 (0.86 to 2.70)	0.14		
Postoperative residual tumor volume [cm^3^]#	1.06 (0.85 to 1.33)	0.60					1.06 (0.82 to 1.37)	0.67
Age at BM diagnosis	1.02 (1.00 to 1.04)	0.055	1.01 (0.99 to 1.04)	0.18	1.02 (0.99 to 1.04)	0.16	1.01 (0.99 to 1.03)	0.23
Preoperative KPS ≥ 70								
- No - Yes	- 0.87 (0.40 to 1.90)	0.70						
BC molecular subtype								
- HER2-/HR- (TN) - HER2 + /HR + - HER2 + /HR- - HER2-/HR +	- 0.42 (0.21 to 0.84) 0.59 (0.27 to 1.31) 0.98 (0.54 to 1.78)	0.015* 0.20 > 0.90						
BC HER2 positivity								
- No - Yes	- 0.49 (0.30 to 0.80)	0.004*	- 0.47 (0.27 to 0.81)	0.006*	- 0.43 (0.25 to 0.75)	0.003*	- 0.50 (0.29 to 0.87)	0.014*
Symptomatic BM								
- No - Yes	- 2.71 (0.98 to 7.54)	0.056	- 3.81 (1.30 to 11.14)	0.014*	- 4.03 (1.36 to 11.95)	0.012*	- 4.55 (1.35 to 15.38)	0.015*
Number of metastatic lesions								
- 1 - 2–5 - > 5	- 1.14 (0.68 to 1.89) 1.29 (0.62 to 2.68)	0.60 0.50	- 0.93 (0.53 to 1.65) 0.69 (0.28 to 1.68)	0.81 0.41	- 1.04 (0.59 to 1.82) 0.95 (0.42 to 2.15)	0.90 0.90	- 0.97 (0.55 to 1.72) 0.80 (0.35 to 1.82)	0.93 0.59
Extracranial metastases at BM diagnosis								
- No - Yes	- 2.21 (1.34 to 3.65)	0.002*	- 2.78 (1.53 to 5.03)	< 0.001*	- 2.81 (1.56 to 5.06)	< 0.001*	- 2.60 (1.44 to 4.70)	0.002*
Time interval between breast cancer and BM diagnosis	0.96 (0.78 to 1.19)	0.70						
Time interval between metastatic disease diagnosis and BM diagnosis	1.14 (0.92 to 1.41)	0.20						
Time interval between BM diagnosis and surgery	0.84 (0.67 to 1.05)	0.12						
Preoperative systemic treatment								
- No - Yes	- 0.94 (0.59 to 1.49)	0.80						
Preoperative radiotherapy								
- No - Yes	- 0.96 (0.42 to 2.22)	> 0.90						

BC, breast cancer; BM, brain metastasis; KPS, Karnofsky Performance Status; HER2, human epidermal growth factor receptor-2; HR, hormone receptor; HR, hazard ratio; TN, triple negative tumor

^#^Extent of resection and postoperative residual tumor volume values were scaled before fitting Cox regression model. *p value < 0.05

The results of the Cox proportional hazard model analysis for intracranial PFS are summarized in Table 3. Similarly to the OS analysis, EOR, GTR achievement, RV, and number of BM lesions were not associated with intracranial PFS in either the univariable or multivariable models. Age and the BC HER2 status were also not associated with intracranial PFS, while the presence of extracranial metastases and symptomatic lesions were independent predictors of shorter intracranial PFS. Subgroup analyses (Supplemental Tables 4–5) further showed no association between EOR, GTR achievement, and RV with intracranial PFS.

**Table 3:** Cox regression analysis of the association between postoperative tumor volume load and intracranial progression-free survival

	Univariable		Multivariable					
	HR (95% CI)	*P* value	HR (95% CI)	*P* value	HR (95% CI)	*P* value	HR (95% CI)	*P* value
Extent of resection (EOR)#	0.88 (0.72 to 1.08)	0.20	0.95 (0.76 to 1.20)	0.67				
Gross-total resection (GTR)								
- Not achieved - Achieved	- 0.88 (0.57 to 1.38)	0.60			1.12 (0.65 to 1.94)	0.68		
Postoperative residual tumor volume [cm^3^]#	1.09 (0.93 to 1.28)	0.30					0.96 (0.79 to 1.15)	0.65
Age at BM diagnosis	0.99 (0.97 to 1.01)	0.2	0.99 (0.97 to 1.01)	0.26	0.99 (0.97 to 1.01)	0.30	0.99 (0.97 to 1.01)	0.23
Preoperative KPS ≥ 70								
- No - Yes	- 1.22 (0.58 to 2.54)	0.60						
BC molecular subtype								
- HER2-/HR- (TN) - HER2 + /HR + - HER2 + /HR- - HER2-/HR +	- 0.61 (0.33 to 1.13) 1.09 (0.54 to 2.19) 0.52 (0.29 to 0.92)	0.12 0.80 0.024*						
BC HER2 positivity								
- No - Yes	- 1.14 (0.74 to 1.76)	0.50	- 1.22 (0.75 to 2.00)	0.42	- 1.22 (0.75 to 2.00)	0.42	- 1.40 (0.85 to 2.33)	0.19
Symptomatic BM								
- No - Yes	- 2.27 (0.99 to 5.22)	0.053	- 2.33 (0.98 to 5.51)	0.055	- 2.41 (1.01 to 5.75)	0.047*	- 2.86 (1.11 to 7.36)	0.029*
Number of metastatic lesions								
- 1 - 2–5 - > 5	- 1.33 (0.84 to 2.12) 1.69 (0.88 to 3.26)	0.20 0.11	- 1.09 (0.65 to 1.82) 1.30 (0.63 to 2.68)	0.74 0.48	- 1.15 (0.68 to 1.95) 1.44 (0.69 to 3.01)	0.61 0.33	- 1.18 (0.70 to 1.97) 1.55 (0.76 to 3.15)	0.54 0.23
Extracranial metastases at BM diagnosis								
- No - Yes	- 1.52 (0.97 to 2.37)	0.065	- 1.84 (1.10 to 3.01)	0.020*	- 1.85 (1.11 to 3.08)	0.020*	- 1.78 (1.06 to 3.00)	0.030*
Time interval between breast cancer and BM diagnosis	0.81 (0.65 to 1.02)	0.068						
Time interval between metastatic disease diagnosis and BM diagnosis	0.82 (0.66 to 1.02)	0.073						
Time interval between BM diagnosis and surgery	0.91 (0.75 to 1.11)	0.30						
Preoperative systemic treatment								
- No - Yes	- 0.98 (0.64 to 1.51)	> 0.90						
Preoperative radiotherapy								
- No - Yes	- 0.67 (0.34 to 1.35)	0.30						

BC, breast cancer; BM, brain metastasis; KPS, Karnofsky Performance Status; HER2, human epidermal growth factor receptor-2; HR, hormone receptor; NA, not applicable; HR, hazard ratio; TN, triple negative tumor

^#^Extent of resection and postoperative residual tumor volume values were scaled before fitting Cox regression model. *p value < 0.05

## Discussion

### Main findings

We conducted a retrospective analysis to evaluate the role of microsurgical tumor burden reduction on OS and intracranial PFS in a homogeneous cohort of patients with BCBM treated at two tertiary brain tumor centers in Switzerland. The observed median OS and intracranial PFS were 22 months (95% CI, 16 to 30) and 8 months (95% CI, 6 to 10), respectively. Across the entire cohort, number of BM lesions, EOR, GTR achievement, and RV showed no association with OS or intracranial PFS. However, in patients without extracranial metastases at BM diagnosis, higher EOR and lower RV were associated with extended OS. Moreover, BC HER2-positive status was strongly associated with prolonged OS, while the presence of extracranial metastases at BM diagnosis and symptomatic BM were correlated with both decreased OS and decreased intracranial PFS.

### Overall survival analysis

The median overall survival of 22 months in our study surpasses that reported in previous studies [12, 20, 21], but aligns with findings from a recently published series of patients primarily treated with SRS [13]. This variance can be attributed to two main factors. First, the enhanced efficacy of contemporary systemic and local treatments within the context of a multimodal care approach [7]. Secondly, the inherent selection bias, arising from the inclusion of patients who were fit enough to undergo surgical treatment and/or radiotherapy. In line with findings from other studies [12, 13, 21–23], we observed a positive association or tendency between longer survival and several factors, including age, BC HER2-positive status, systemic disease control, and asymptomatic BM. However, we did not detect a significant decrease in OS among patients with multiple lesions [24]. Notably, 88% of the patients included in our study had fewer than 5 lesions, and 52% had only one lesion, therefore hindering a meaningful comparison with subgroups characterized by a higher lesion count.

The literature on the association of EOR, GTR achievement, and RV with OS in patients with BM disease is conflicting [5, 13, 23]. While some studies [23, 25–28] have documented a positive association between greater EOR or smaller postoperative tumor volume and longer survival, others [24, 29–32] reported no significant association. However, all these studies included heterogeneous populations, with only a small proportion having BCBM. It has been suggested that the association of residual tumor with OS is linked to better local disease control [33] and to a faster tapering of steroids after surgery and therefore an increased therapeutic efficacy of subsequent treatment with e.g. immune checkpoint inhibitors [34]. Several factor might explain the lack of association between EOR, GTR achievement, RV, and OS in our cohort. First, the relatively small sample size may have limited the power to detect significant associations. Secondly, postoperative systemic treatment for BMBC primarily focuses on HER2 targeted therapy and endocrine therapy, with immunotherapy used infrequently. Thirdly, the substantial number of patients presenting with extracranial metastases at the time of BM diagnosis, likely indicating uncontrolled systemic disease, which could have diminished the positive impact of controlling intracranial disease on OS. Consistent with this, a significant association of EOR and RV with OS was found in the subgroup of patients without extracranial metastases at BM diagnosis. Lastly, the multimodal treatment approach, including the widespread use of postoperative radiotherapy (84%) and additional local interventions (44%) may have significantly influenced the intracranial disease course after microsurgical resection. The standard use of postoperative radiotherapy may have reduced residual tumors, while additional local interventions may have improved the control of BM lesions not addressed by surgery or newly presenting BM lesions [35].

Interestingly, although we did not find a significant association between postoperative tumor volume and OS, the predominant cause of death in our cohort was intracranial disease progression (43% of patients). This finding is similar to a recently published study in a cohort of patients with resected BM from non-small cell lung cancer [24], but it is in contrast to the results from older studies on patients who underwent operations for BM from various primary tumors [29, 36–38], which reported that most patients died of systemic disease progression. These differences could be explained by the increasing use of systemic treatments (75% of patients), which have become more efficient for systemic disease control, but still have limited penetration into the brain. Although this aspect warrants further investigation, it underscores the importance of regular follow-up of patients with BCBM after surgery in order to ensure timely assessment of intracranial progression and evaluate the necessity for additional local interventions to achieve cerebral disease control.

### Intracranial progression-free survival analysis

The median intracranial PFS of 8 months aligns with findings from recent studies in patients treated with surgical resection for BM from various primary tumors [24, 39]. Among patients experiencing cerebral progression, only 12% exhibited local recurrence, while 88% had distant recurrence or a combination of local and distant recurrence. This indicates that most progression stemmed from the emergence of new lesions or the growth of unresected ones. However, no association between EOR, GTR achievement, RV, and intracranial PFS was found, while presence of extracranial metastases and symptomatic BM lesions correlated with shorter intracranial PFS. Similar to OS, the absence of association between postoperative tumor volume and intracranial progression could be attributed to several factors. These include the relatively small sample size of the cohort, the multimodal treatment approach, or the substantial number of patients with extracranial metastases at BM diagnosis. The positive impact of additional local interventions during follow-up is also evident in the discrepancy between median intracranial PFS and median OS, highlighting that prolonged OS can still be achieved even after progression.

### The role of microsurgical resection

The main objectives of surgical resection for BM are to relieve pressure on critical brain regions, alleviate neurological symptoms, achieve local disease control, and obtain tissue samples for histopathological analysis. We recently reported that microsurgical resection is a safe therapeutic option for treating BM with low postoperative morbidity [40, 41]. However, its role on survival outcomes within a multimodal treatment approach remains to be fully elucidated, given that existing studies often lack well-defined, homogeneous cohorts of BM patients. A recent study from our group [8] has shown the positive association between surgical tumor burden reduction and improved OS in melanoma BM. Nevertheless, our current analysis did not identify such an association, suggesting that the impact of surgical tumor burden reduction on survival outcomes may vary depending on factors such as the primary tumor type and the efficacy of systemic treatments.

### Limitations

This study has several limitations. First, the relatively small sample size may have limited the detection of significant associations. Second, focusing exclusively on surgically treated BCBM patients introduces selection bias, limiting the generalizability of our results to the entire BCBM population. Third, the retrospective nature of the analysis and the lack of a systematic imaging follow-up protocol, along with follow-ups at other institutions, could have introduced bias in the analysis of intracranial progression-free survival (PFS). Additionally, variations in pre- and postoperative intracranial tumor volumetric assessments using CT or a combination of MRI and CT could potentially introduce errors, despite some comparability between these two techniques has been shown [42, 43]. Finally, the clinical practice setting of this analysis, while allowing exploration of surgical treatment within a multimodal approach, limits our ability to draw conclusions about the benefits of surgery compared to other treatments, such as radiotherapy.

## Conclusions

In the era of modern systemic and local treatments integrated within a multimodal care approach, we show extended OS among surgically treated patients with BCBM. Breast cancer HER2-positive status was associated with longer OS, whereas the presence of extracranial metastases, and symptomatic BM lesions were associated with both reduced OS and intracranial PFS. Although a more aggressive resection aimed at minimizing residual tumor volume did not show a significant association with OS or intracranial PFS across the entire cohort, it seemed to be linked to prolonged OS in patients without extracranial metastases. Additional research is required to further elucidate the role of surgical tumor burden reduction in this specific patient group. Meanwhile, microsurgery should prioritize symptom alleviation and obtaining tumor tissue for histopathological examination.

### Supplementary Information

Below is the link to the electronic supplementary material.

Supplementary file1 (DOCX 191 KB)

## Data Availability

Anonymized datasets are available upon reasonable request.
