# Supplementary material for: Role of microsurgical tumor burden reduction in patients with breast cancer brain metastases considering molecular subtypes: a two-center volumetric survival analysis
Source: J Neurooncol. 2024 Jun 3;169(2):379–90. doi: 10.1007/s11060-024-04728-w (PMC11341656; doi:10.1007/s11060-024-04728-w)
Supplement: Supplementary file 1 — Supplementary file1 (DOCX 191 KB) [file 11060_2024_4728_MOESM1_ESM.docx]

## SUPPLEMENTAL TABLES AND FIGURES

|  | **N = 101** |
| --- | --- |
| **Postoperative radiotherapy (%)**   - SRS - WBRT - SRS + WBRT - None - NA | 46 (45)  27 (27)  12 (12)  10 (10)  6 (6) |
| **Additional postoperative local interventions (%)**   - Yes - No | 44 (44)  57 (56) |
| **Postoperative systemic treatment (%)**   - Yes - No - NA | 76 (75)  21 (21)  4 (4) |
| **Postoperative cytotoxic chemotherapy (%)**   - Yes - No - NA | 45 (45)  52 (51)  4 (4) |
| **Postoperative HER2+ targeted therapy (%)**   - Yes - No - NA | 36 (36)  61 (60)  4 (4) |
| **Postoperative endocrine therapy (%)**   - Yes - No - NA | 34 (34)  63 (62)  4 (4) |
| **Postoperative CDK4/6 targeted therapy (%)**   - Yes - No - NA | 1 (1)  96 (95)  4 (4) |
| **Postoperative immune checkpoint inhibitor therapy (%)**   - Yes - No - NA | 2 (2)  95 (94)  4 (4) |
| **Postoperative PARP inhibitor therapy (%)**   - Yes - No - NA | 3 (3)  94 (93)  4 (4) |

**Supplemental Table 1. Postoperative treatments.**

CDK4/6, cyclin-dependent kinase 4/6; HER2, human epidermal growth factor receptor-2; NA, not available; PARP, poly (ADP-ribose) polymerase; SRS, stereotactic radiosurgery; WBRT, whole-brain radiotherapy.

|  | **Breast cancer HER2 positive status** | | **Breast cancer HER2 negative status** | |
| --- | --- | --- | --- | --- |
|  | HR (95% CI) | *P* value | HR (95% CI) | *P* value |
| **Extent of resection (EOR)#** | 1.01 (0.61 to 1.66) | >0.90 | 0.94 (0.69 to 1.30) | 0.70 |
| **Gross-total resection (GTR)**  - Not achieved  - Achieved | -  1.05 (0.61 to 1.66) | >0.90 | -  1.52 (0.81 to 2.85) | 0.20 |
| **Postoperative residual tumor volume [cm^3^]#** | 1.12 (0.73 to 1.72) | 0.60 | 1.16 (0.86 to 1.56) | 0.30 |

**Supplemental Table 2. Cox regression analysis of the association between postoperative tumor volume load and overall survival stratified by breast cancer HER2 status.**

BM, brain metastasis; HER2, human epidermal growth factor receptor-2; HR, hazard ratio.

Only univariable analysis were performed. #Extent of resection and postoperative residual tumor volume values were scaled before fitting Cox regression model. *p value < 0.05.

|  | **Extracranial disease positive status** | | **Extracranial disease negative status** | |
| --- | --- | --- | --- | --- |
|  | HR (95% CI) | *P* value | HR (95% CI) | *P* value |
| **Extent of resection (EOR)#** | 1.29 (0.92 to 1.80) | 0.14 | 0.62 (0.41 to 0.94) | **0.024*** |
| **Gross-total resection (GTR)**  - Not achieved  - Achieved | -  0.96 (0.51 to 1.83) | >0.90 | -  1.32 (0.58 to 3.01) | 0.50 |
| **Postoperative residual tumor volume [cm^3^]#** | 0.94 (0.67 to 1.30) | 0.70 | 1.57 (1.03 to 2.39) | **0.035*** |

**Supplemental Table 3. Cox regression analysis of the association between postoperative tumor volume load and overall survival stratified by the presence of extracranial metastases at time of brain metastasis diagnosis.**

BM, brain metastasis; HER2, human epidermal growth factor receptor-2; HR, hazard ratio.

Only univariable analysis were performed. #Extent of resection and postoperative residual tumor volume values were scaled before fitting Cox regression model. *p value < 0.05.

|  | **Breast cancer HER2 positive status** | | **Breast cancer HER2 negative status** | |
| --- | --- | --- | --- | --- |
|  | HR (95% CI) | *P* value | HR (95% CI) | *P* value |
| **Extent of resection (EOR)#** | 0.93 (0.68 to 1.27) | 0.70 | 0.84 (0.62 to 1.14) | 0.30 |
| **Gross-total resection (GTR)**  - Not achieved  - Achieved | -  0.74 (0.35 to 1.53) | 0.40 | -  1.13 (0.62 to 2.07) | 0.70 |
| **Postoperative residual tumor volume [cm^3^]#** | 1.00 (0.76 to 1.32) | >0.90 | 1.23 (0.95 to 1.60) | 0.11 |

**Supplemental Table 4. Cox regression analysis of the association between postoperative tumor volume load and intracranial progression-free survival stratified by breast cancer HER2 status.**

BM, brain metastasis; HER2, human epidermal growth factor receptor-2; HR, hazard ratio.

Only univariable analysis were performed. #Extent of resection and postoperative residual tumor volume values were scaled before fitting Cox regression model. *p value < 0.05.

|  | **Extracranial disease positive status** | | **Extracranial disease negative status** | |
| --- | --- | --- | --- | --- |
|  | HR (95% CI) | *P* value | HR (95% CI) | *P* value |
| **Extent of resection (EOR)#** | 0.84 (0.64 to 1.09) | 0.20 | 0.95 (0.65 to 1.37) | 0.90 |
| **Gross-total resection (GTR)**  - Not achieved  - Achieved | -  0.92 (0.51 to 1.67) | 0.80 | -  1.05 (0.51 to 2.17) | 0.90 |
| **Postoperative residual tumor volume [cm^3^]#** | 1.06 (0.86 to 1.30) | 0.60 | 1.27 (0.87 to 1.86) | 0.20 |

**Supplemental Table 5. Cox regression analysis of the association between postoperative tumor volume load and intracranial progression-free survival stratified by the presence of extracranial metastases at time of brain metastasis diagnosis.**

BM, brain metastasis; HER2, human epidermal growth factor receptor-2; HR, hazard ratio.

Only univariable analysis were performed. #Extent of resection and postoperative residual tumor volume values were scaled before fitting Cox regression model. *p value < 0.05.


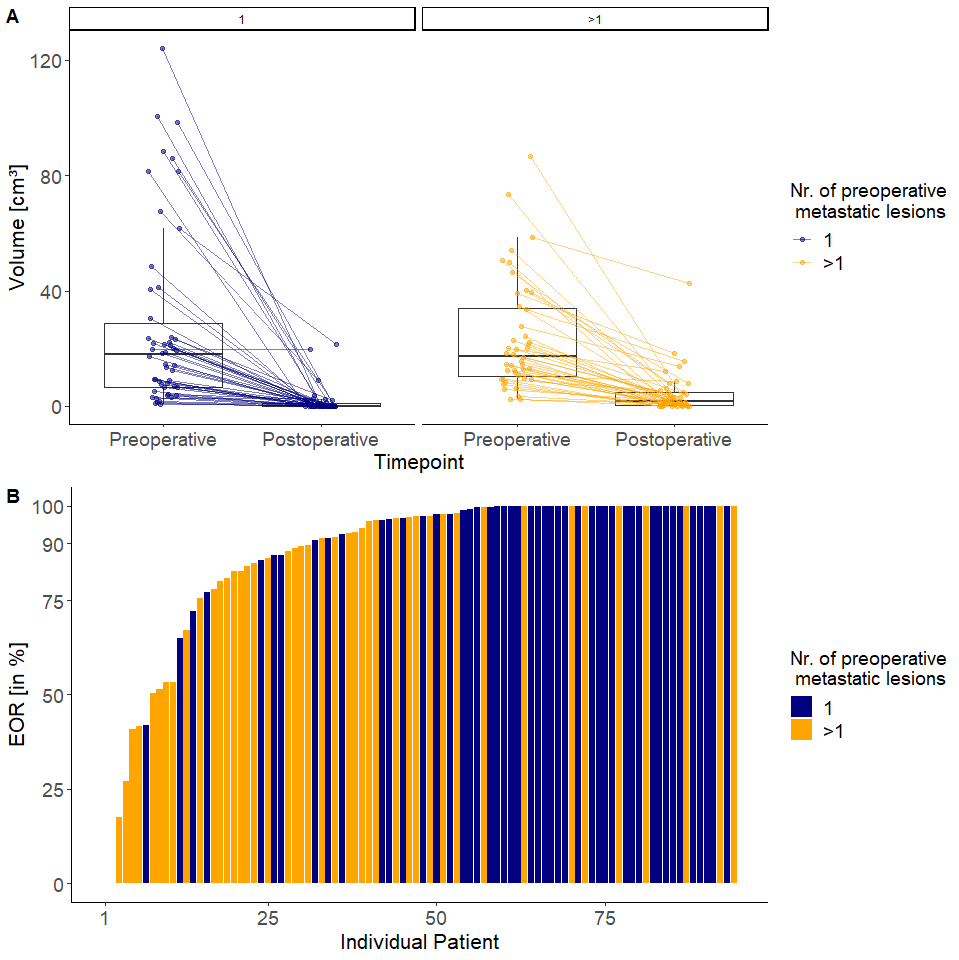


**Supplemental Figure 1. Extent of resection and residual tumor volume.**

(A) Pre- and postoperative total intracranial tumor volume as well as extent of resection (B) stratified by the number of metastatic lesions (1 or >1) are illustrated. EOR, extent of resection.


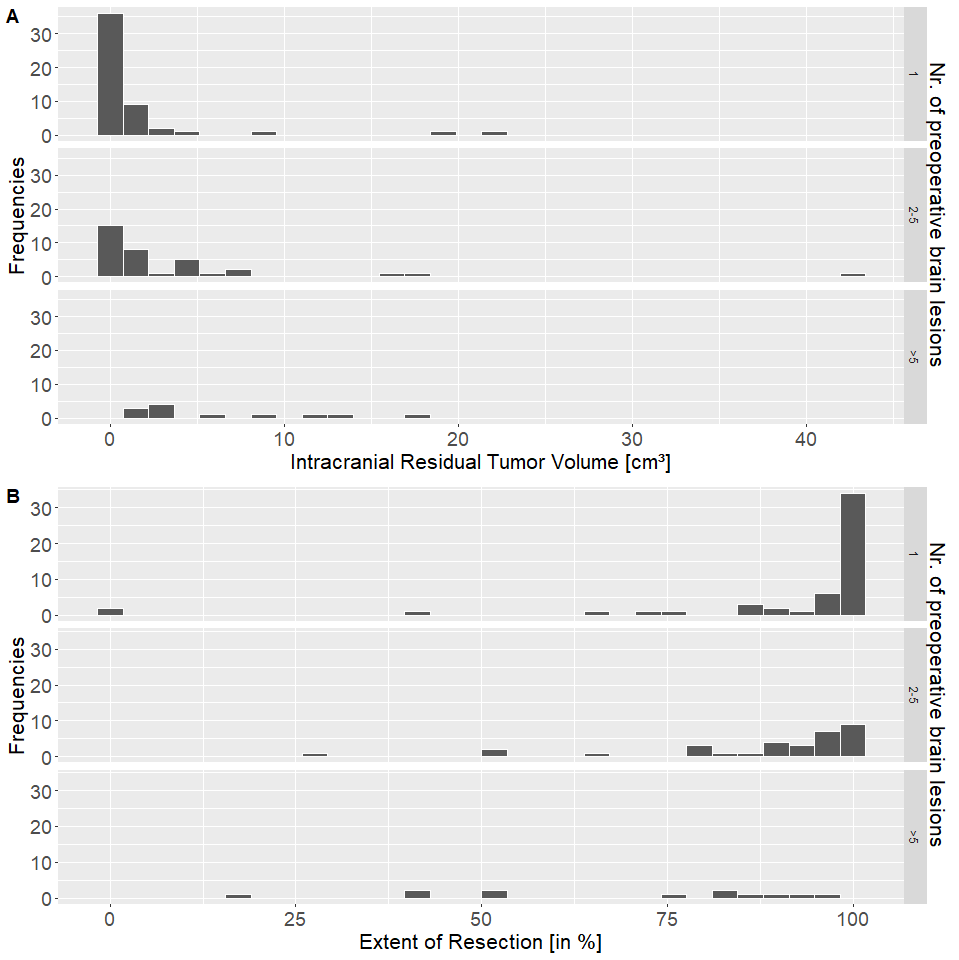


**Supplemental Figure 2. Residual tumor volume and extent of resection according to the preoperative number of metastatic lesions.**

(A) Postoperative residual intracranial tumor volume in cm^3^ and extent of resection (B) stratified by the number of metastatic lesion(s): 1, 2-5, >5. EOS, extent of resection.


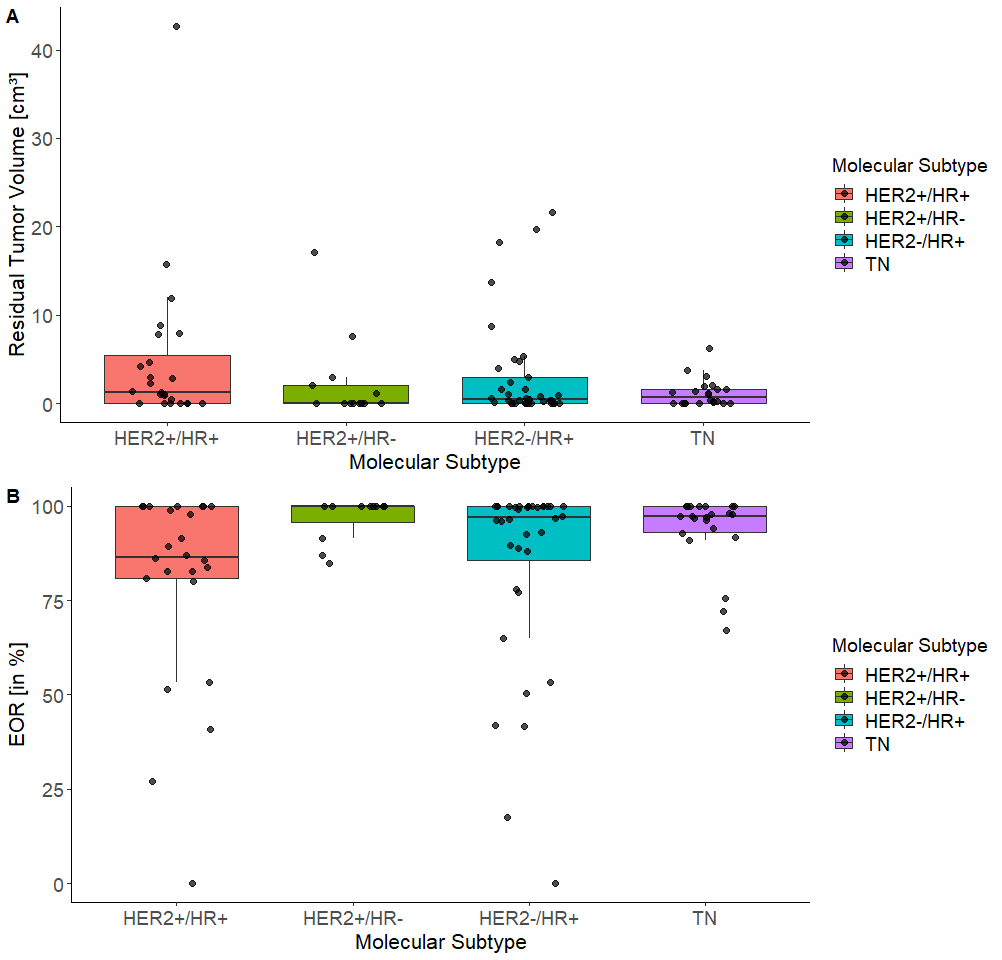


**Supplemental Figure 3. Residual tumor volume and extent of resection according to breast cancer molecular subtype.**

(A) Postoperative intracranial residual tumor volume and extent of resection (EOR, B) stratified by breast cancer molecular subtype. BC, breast cancer; EOR, extent of resection; HER2, human epidermal growth factor receptor-2; HR, hormone receptor; TN, triple-negative.


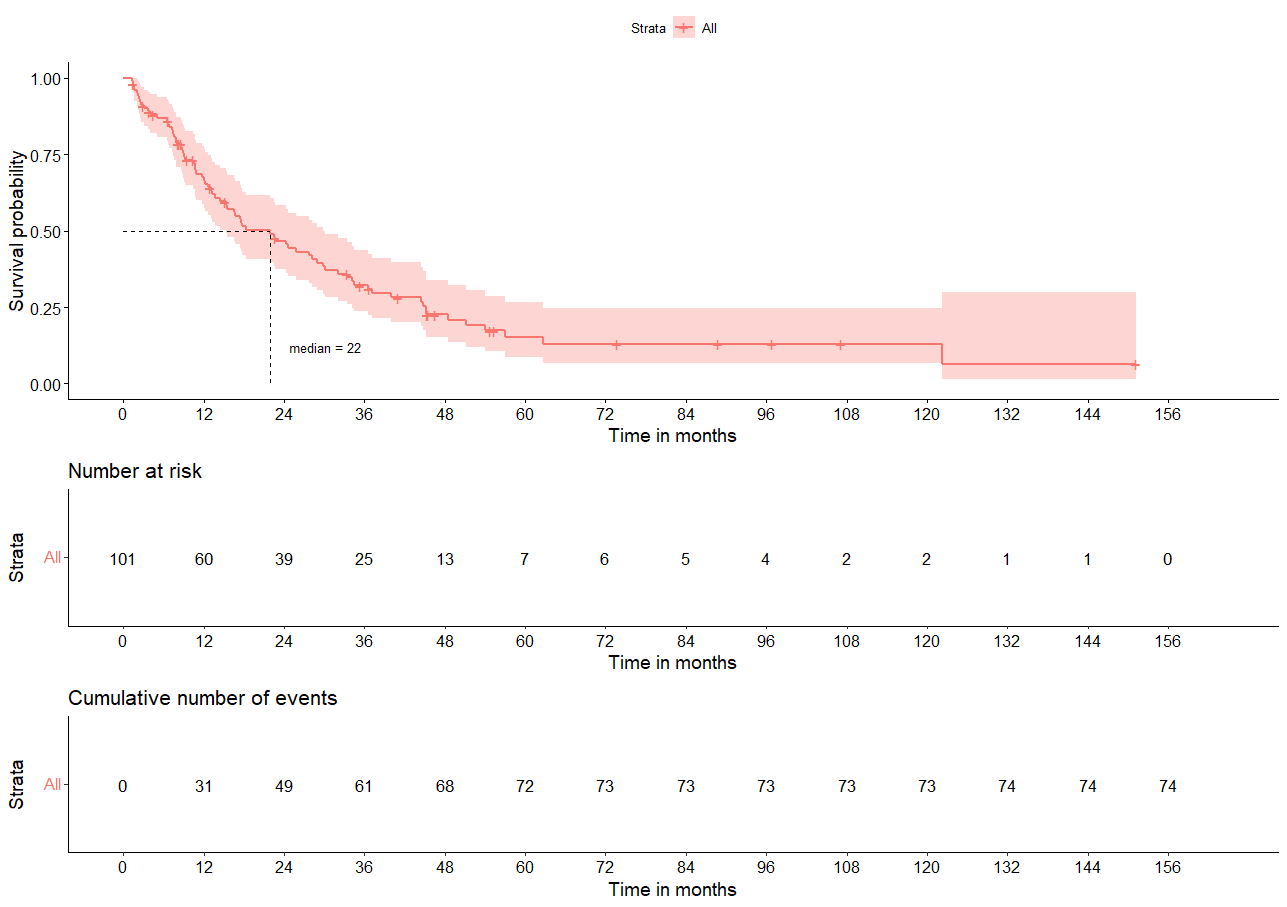


**Supplemental Figure 4. Overall survival in the study cohort.**

Time is reported in months from surgery.


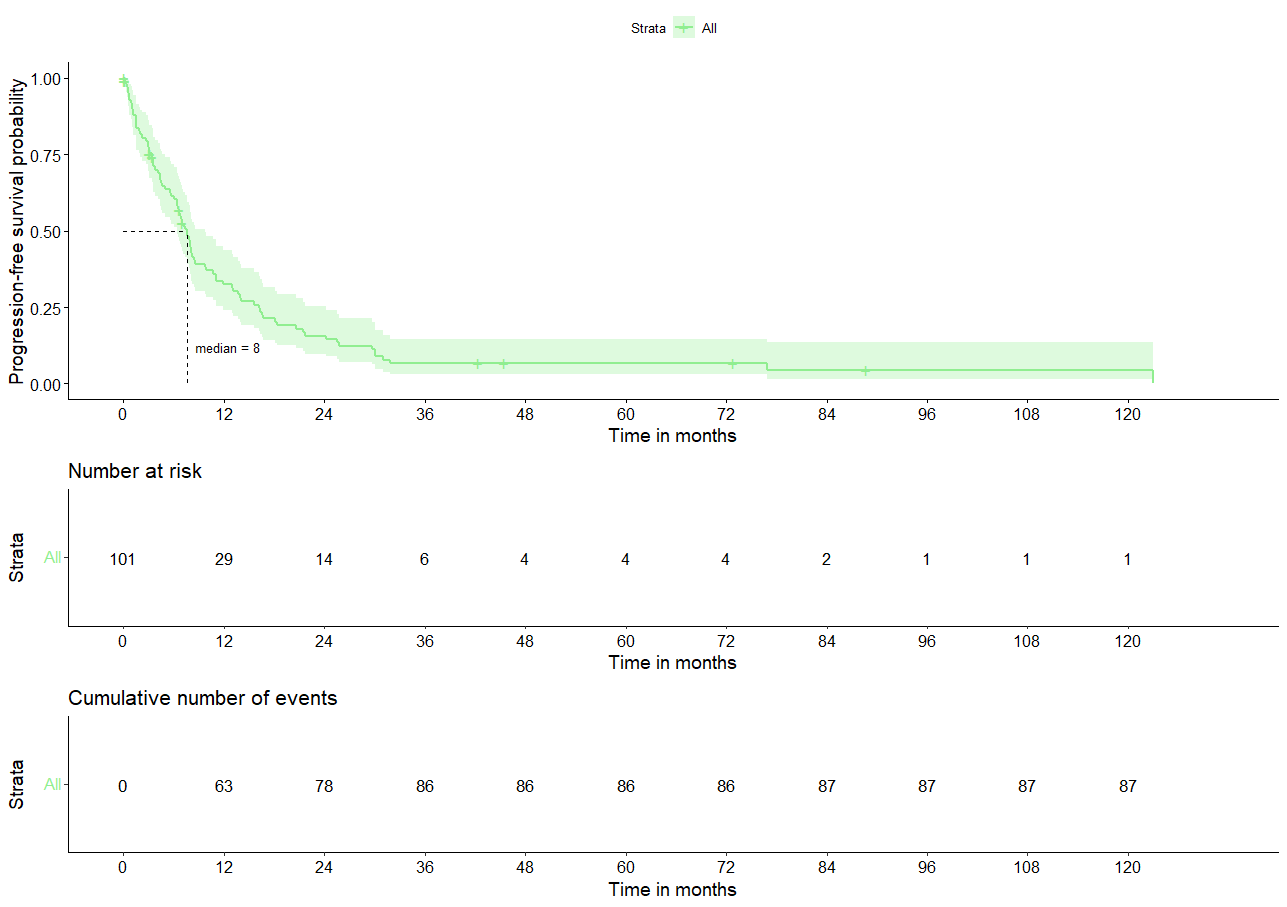


**Supplemental Figure 5. Intracranial progression-free survival in the study cohort.**

Time is reported in months from surgery.

**
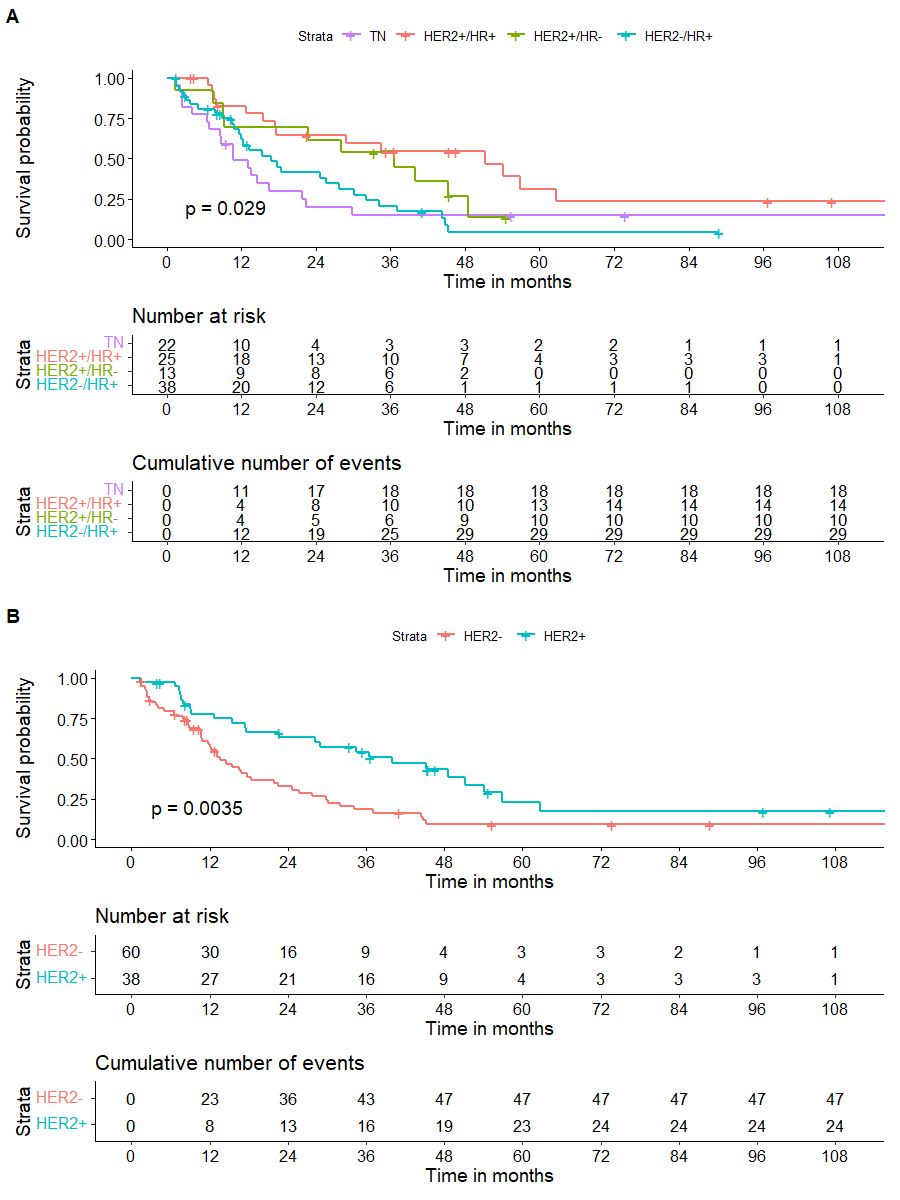
**

**Supplemental Figure 6. Overall survival according to the breast cancer molecular subtype.**

Kaplan-Meier curve with log-rank statistic of patients stratified by the breast cancer molecular subtype (A) and by breast cancer HER2 positivity (B). Time is reported in months from surgery; Follow-up time is clipped at 9 years for better readability. BC, breast cancer; HER2, human epidermal growth factor receptor-2; HR, hormone receptor.


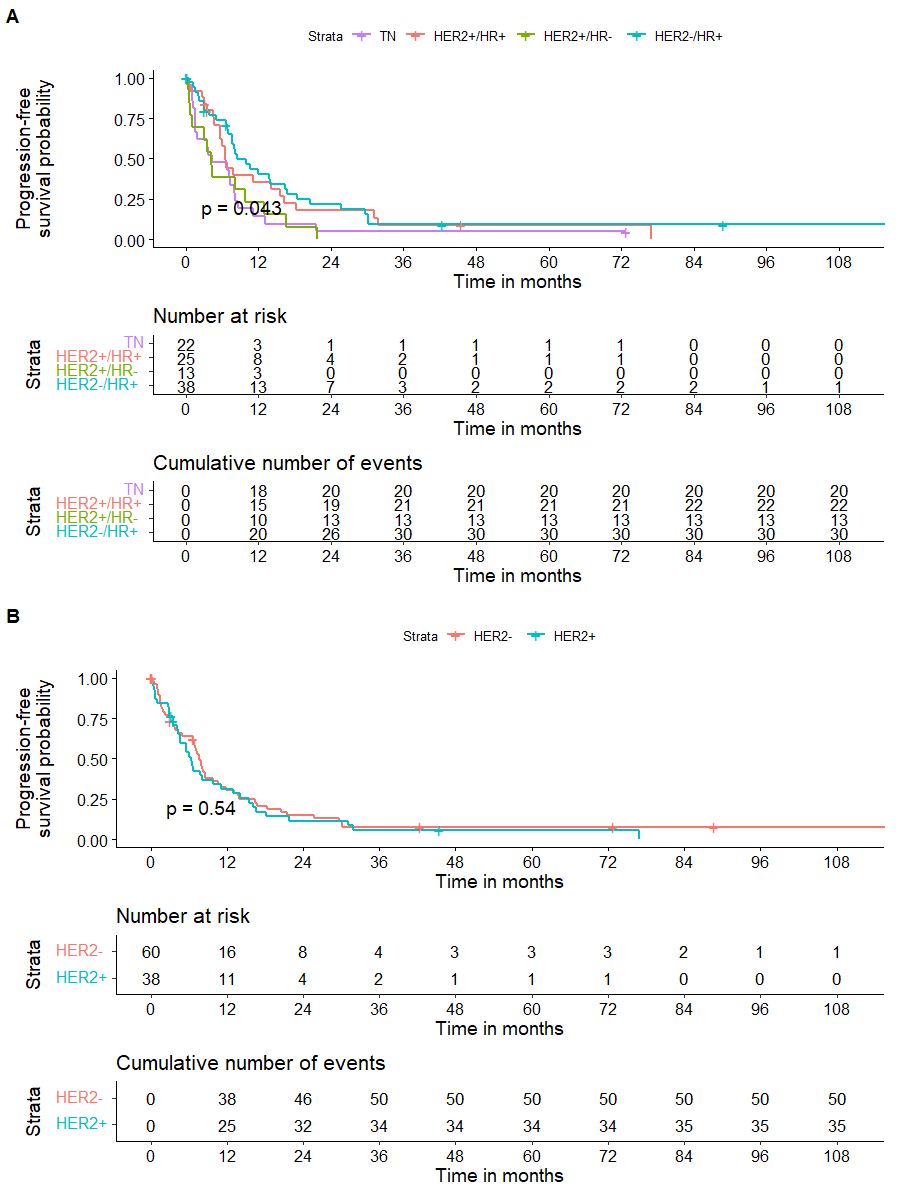


**Supplemental Figure 7. Intracranial progression-free survival according to the breast cancer molecular subtype.**

Kaplan-Meier curve with log-rank statistic of patients stratified by the breast cancer molecular subtype (A) and by breast cancer HER2 positivity (B). Time is reported in months from surgery; Follow-up time is clipped at 9 years for better readability. BC, breast cancer; HER2, human epidermal growth factor receptor-2; HR, hormone receptor.
